# Supplementary material for: Early Enriched Environment Prevents Epigenetic p11 Gene Changes Induced by Adulthood Stress in Mice
Source: Int J Mol Sci. 2021 Feb 15;22(4):1928. doi: 10.3390/ijms22041928 (PMC7919643; doi:10.3390/ijms22041928)
Supplement: Supplementary file 1 [file ijms-22-01928-s001.pdf]

Supplementary Material

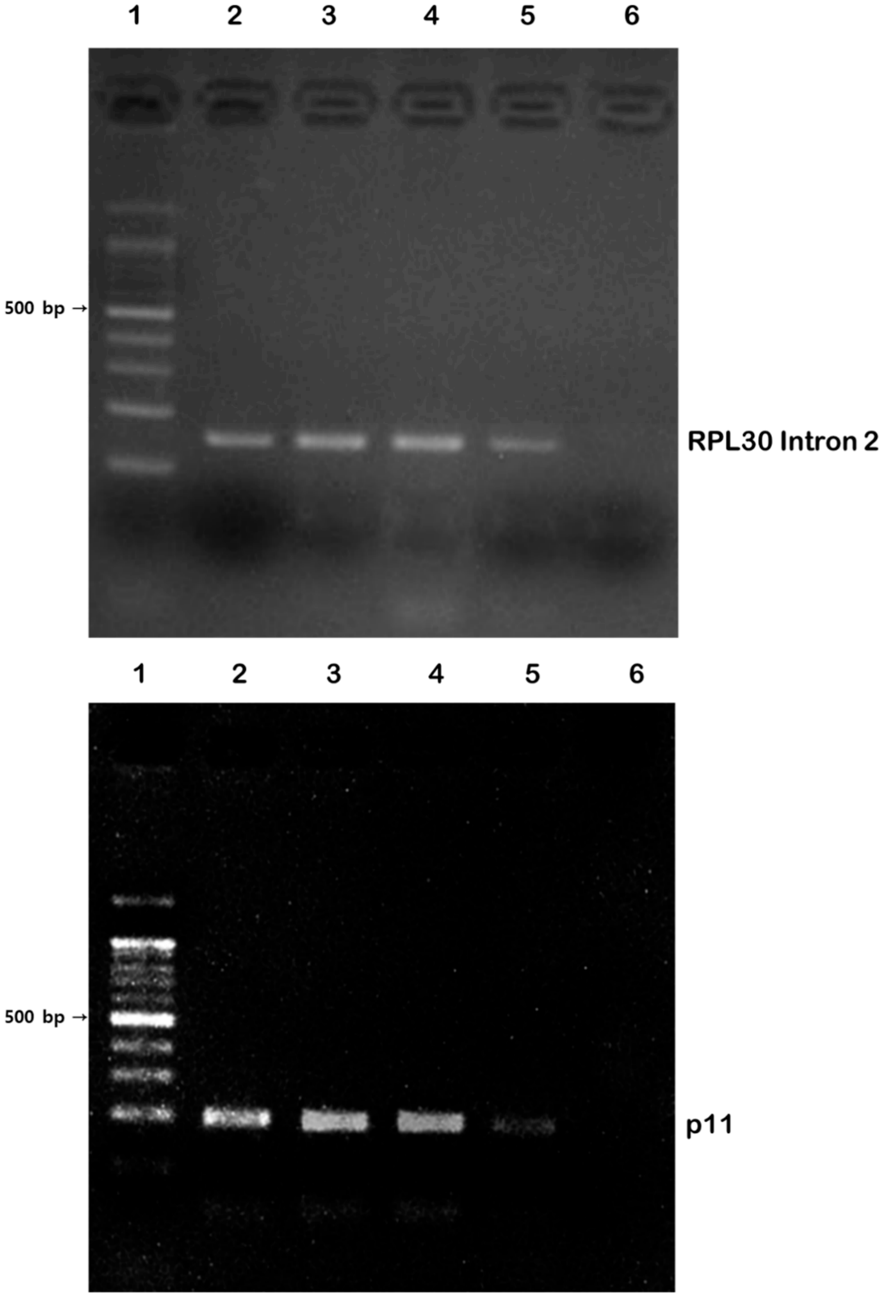

Figure S1. Antibody specificity in the chromatin immunoprecipitation (ChIP) assay. ChIP was performed using digested chromatin from mouse hippocampal tissue and the indicated antibodies. Quantitative real-time polymerase chain reaction (qRT-PCR) was used to analyze purified DNA with the SimpleChIP® Mouse RPL30 Intron 2 Promoter Primers #7015 (control primer set; upper bands, 159 base-pairs) and p11 promoter primers (lower bands, 189 base-pairs). qRT-PCR products were observed for each primer set in the input sample (lane 2) and various ChIP samples (lanes 3–5) but not in the normal rabbit IgG ChIP sample (lane 6). PCR products were confirmed by electrophoresis with a 2% agarose gel. Lane 1: Size marker (100 base-pair ladder, EBM-1001; Elpisbio); Lane 2: Positive control; Input sample; Lane 3: AcH3 (K9/K14; 06-599; Millipore-Sigma); Lane 4: H3K4me3 (ab8580; Abcam); Lane 5: H3K27me3 (ab6002; Abcam); Lane 6: Negative control; Normal rabbit IgG (#2729; Cell signaling).
